# Role of the prorenin receptor in endometrial cancer cell growth

**DOI:** 10.18632/oncotarget.28224

**Published:** 2022-04-01

**Authors:** Jacinta H. Martin, Riazuddin Mohammed, Sarah J. Delforce, David A. Skerrett-Byrne, Celine Corbisier de Meaultsart, Juhura G. Almazi, Andrew N. Stephens, Nicole M. Verrills, Evdokia Dimitriadis, Yu Wang, Eugenie R. Lumbers, Kirsty G. Pringle

**Affiliations:** ^1^School of Biomedical Sciences and Pharmacy, Priority Research Centre for Reproductive Science, Mothers and Babies Research Centre, Hunter Medical Research Institute, University of Newcastle, Newcastle, New South Wales, Australia; ^2^School of Environmental and Life Sciences, Priority Research Centre for Reproductive Science, Hunter Medical Research Institute, University of Newcastle, Newcastle, New South Wales, Australia; ^3^School of Biomedical Sciences and Pharmacy, Priority Research Centre for Cancer Research, Innovation and Translation, Hunter Medical Research Institute, University of Newcastle, Newcastle, New South Wales, Australia; ^4^School of Life Sciences, Faculty of Science, University of Technology Sydney, Ultimo, New South Wales, Australia; ^5^Hudson Institute of Medical Research, Clayton, Victoria, Australia; ^6^Department of Molecular and Translational Sciences, Monash University, Clayton, Victoria, Australia; ^7^Department of Obstetrics and Gynaecology, Gynaecology Research Centre, The Women’s Hospital, University of Melbourne, Melbourne, Victoria, Australia; ^*^These authors contributed equally to this work

**Keywords:** (P)RR, ATP6AP2, endometrial cancer, cellular viability, cellular proliferation

## Abstract

Endometrial cancer is the most diagnosed gynecological malignancy. Despite numerous scientific advances, the incidence and mortality rate of endometrial cancer continues to rise. Emerging evidence suggests a putative role of the (pro)renin receptor ((P)RR), in the ontogenesis of endometrial cancer. The (P)RR is implicated in breast cancer and pancreatic carcinoma pathophysiology by virtue of its role in proliferation, angiogenesis, fibrosis, migration and invasion. Thus, we aimed to investigate the functional role of the (P)RR in human endometrial cancer.

We employed an siRNA-mediated knockdown approach to abrogate (P)RR expression in the endometrial epithelial cell lines; Ishikawa, AN3CA and HEC-1-A and examined cellular proliferation and viability. We also carried out a sophisticated proteomic screen to explore potential pathways via which the (P)RR is acting in endometrial cancer physiology.

These data confirmed that the (P)RR is critical for endometrial cancer development, contributing to both its proliferative capacity and in the maintenance of cell viability. This is likely mediated through proteins such as MGA, SLC4A7, SLC7A11 or DHRS2, which were reduced following (P)RR knockdown.

These putative protein interactions/pathways, which rely on the presence of the (P)RR, are likely to contribute to endometrial cancer progression and could therefore, represent several novel therapeutic targets for endometrial cancer.

## INTRODUCTION

Endometrial cancer is the most common gynecological malignancy in the United States [1]. Strikingly, endometrial cancer is the only gynecological cancer with an increasing incidence and mortality rate, with the estimated 5-year survival rate decreasing from 88% in 1975 to 81% in 2020 [2].

The (pro)renin receptor ((P)RR), has been implicated in the ontogenesis of several cancers including breast and pancreatic ductal adenocarcinoma [3, 4]. We have shown that the (P)RR is overexpressed in human endometrial cancer tissues compared with adjacent unaffected endometrium [5]. This is perhaps not surprising given that the (P)RR promotes angiogenesis, proliferation and migration [5], all of which are involved in tumourigenesis and the development and progression of endometrial cancer.

The (P)RR is a single transmembrane domain receptor [6] encoded by the ATPase H(+)-transporting lysosomal accessory protein 2 (*ATP6AP2*) gene, located on the X chromosome. The (P)RR activates several pathways that stimulate proliferation and cellular viability. First, the (P)RR can bind both renin and prorenin [6]. Binding of prorenin to the (P)RR stimulates a conformational change in prorenin, displacing the pro-segment from the molecule’s catalytic cleft making it accessible to angiotensinogen (AGT) [7], thereby activating the renin-angiotensin system (RAS). AGT is then cleaved to form angiotensin (Ang) I and sequentially converted to Ang II by angiotensin-converting enzyme (ACE). Ang II can act via either the angiotensin II type 1 (AT1R; proliferative and pro-angiogenic) or type 2 receptors (AT2R; anti-proliferative and anti-angiogenic).

Second, and independent of the actions of the RAS, binding of prorenin to the (P)RR can mediate the translocation of promyelocytic leukemia zinc finger protein (PLZF) to the nucleus, activating the p85α subunit of phosphatidylinositol-3 kinase (PI3K-p85α), directly triggering cellular proliferation and enhancing cellular viability [8]. The (P)RR can also activate the Wnt/β-catenin signaling pathway, since Wnt ligands are able to bind to the frizzled/low-density lipoprotein receptor related protein complex (FZD/LRP6), which is internalized by V-ATPases such as the (P)RR [9, 10]. Finally, the (P)RR can directly enhance cell proliferation by phosphorylating extracellular signal-regulated kinases 1/2 (ERK 1/2) and mitogen activated protein kinases (MAPK) [11] and, in turn, activating transforming growth factor (TGF)-β1 [3, 10, 12]. Overexpression of the (P)RR in endometrial cancer, could therefore facilitate the rapid growth and spread of this malignancy. In support of this hypothesis, several studies have shown that siRNAs directed against the (P)RR reduce cell proliferation in breast carcinoma cell lines [3], inhibit ERK 1/2 activation in human vascular smooth muscle cells and mesangial cells [13, 14] and reduce tumour growth in a mouse model of pancreatic ductal adenocarcinoma [4].

In view of this compelling data, we aimed to investigate the functional role of (P)RR in human endometrial cancer progression and development. We employed an siRNA-mediated approach to abrogate (P)RR expression in three endometrial epithelial cancer cell lines (Ishikawa, AN3CA and HEC-1-A) to investigate the role of this protein in endometrial cancer cell proliferation and viability. These analyses were carried out alongside a sophisticated proteomic approach, to gain a global overview of the potential role and interactions of the (P)RR in endometrial cancer biology.

## RESULTS

### mRNA and protein expression of ATP6AP2 in Ishikawa, AN3CA and HEC-1-A cells

**Figure 1:**
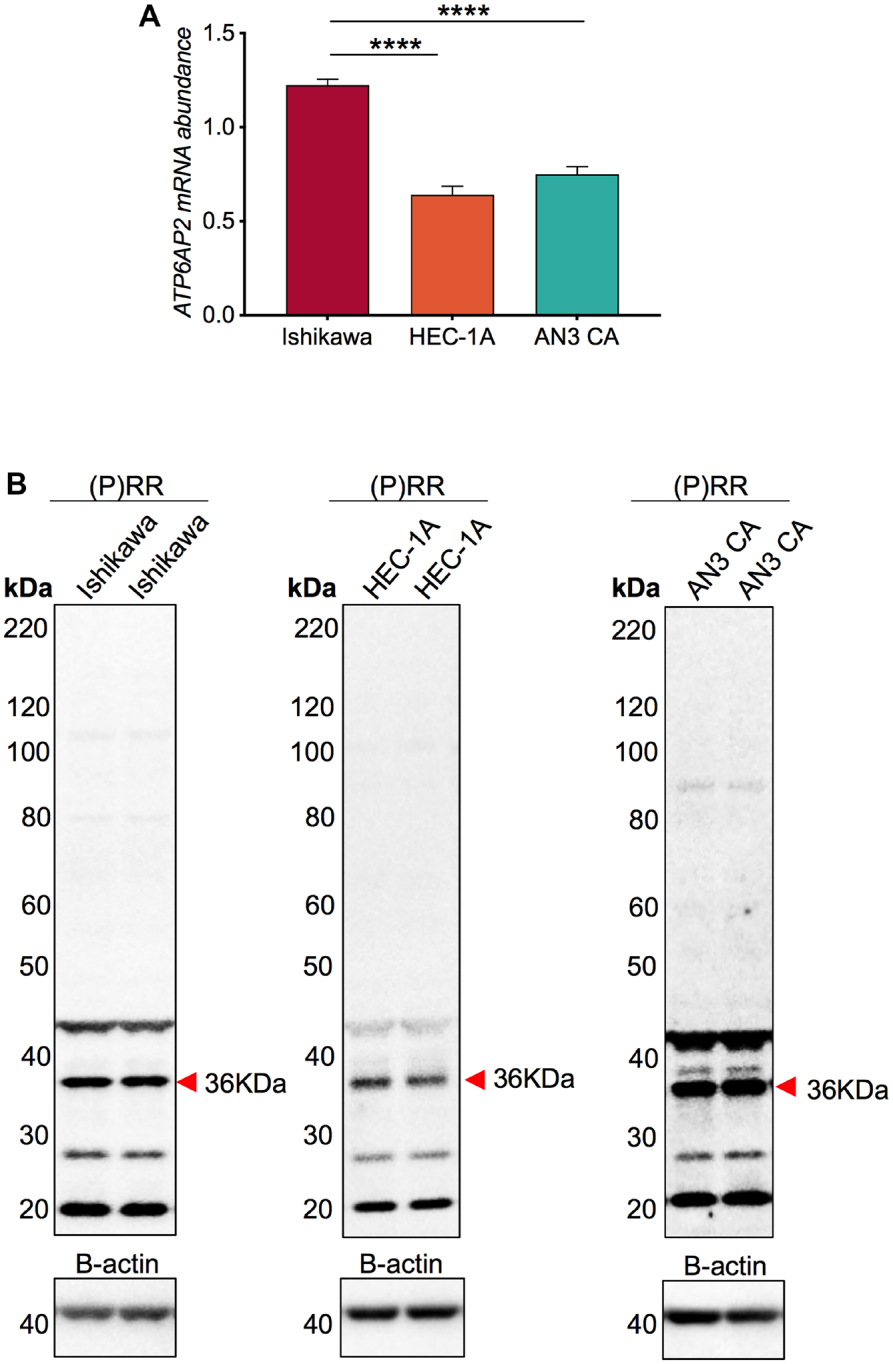
ATP6AP2 mRNA and protein levels in the endometrial epithelial cancer cell lines Ishikawa, AN3CA and HEC-1-A. (**A**) Messenger RNA expression of *ATP6AP2* relative to a human term placenta control. (**B**) Immunoblotting on RIPA extracted cell lysates confirmed the expression of the (pro)renin receptor ((P)RR) protein product (36kDA; red arrow). Mean ± SEM values are plotted in histograms. ^***^ *P* < 0.001 and ^****^ *P* < 0.0001.

*ATP6AP2* mRNA was expressed in each of the three cell lines. Notably, the highest relative expression was detected in Ishikawa cells, which was 2-fold higher than the AN3CA (*P* < 0.0001) and the HEC-1-A cell lines (*P* < 0.0001; Figure 1A). Complementary immunoblotting confirmed that a dominant protein band corresponding to the predicted size for the (P)RR, 36kDA, was detected for each cell line (Figure 1B).

### siRNA knockdown of ATP6AP2 in Ishikawa, AN3CA and HEC-1-A cells

**Figure 2:**
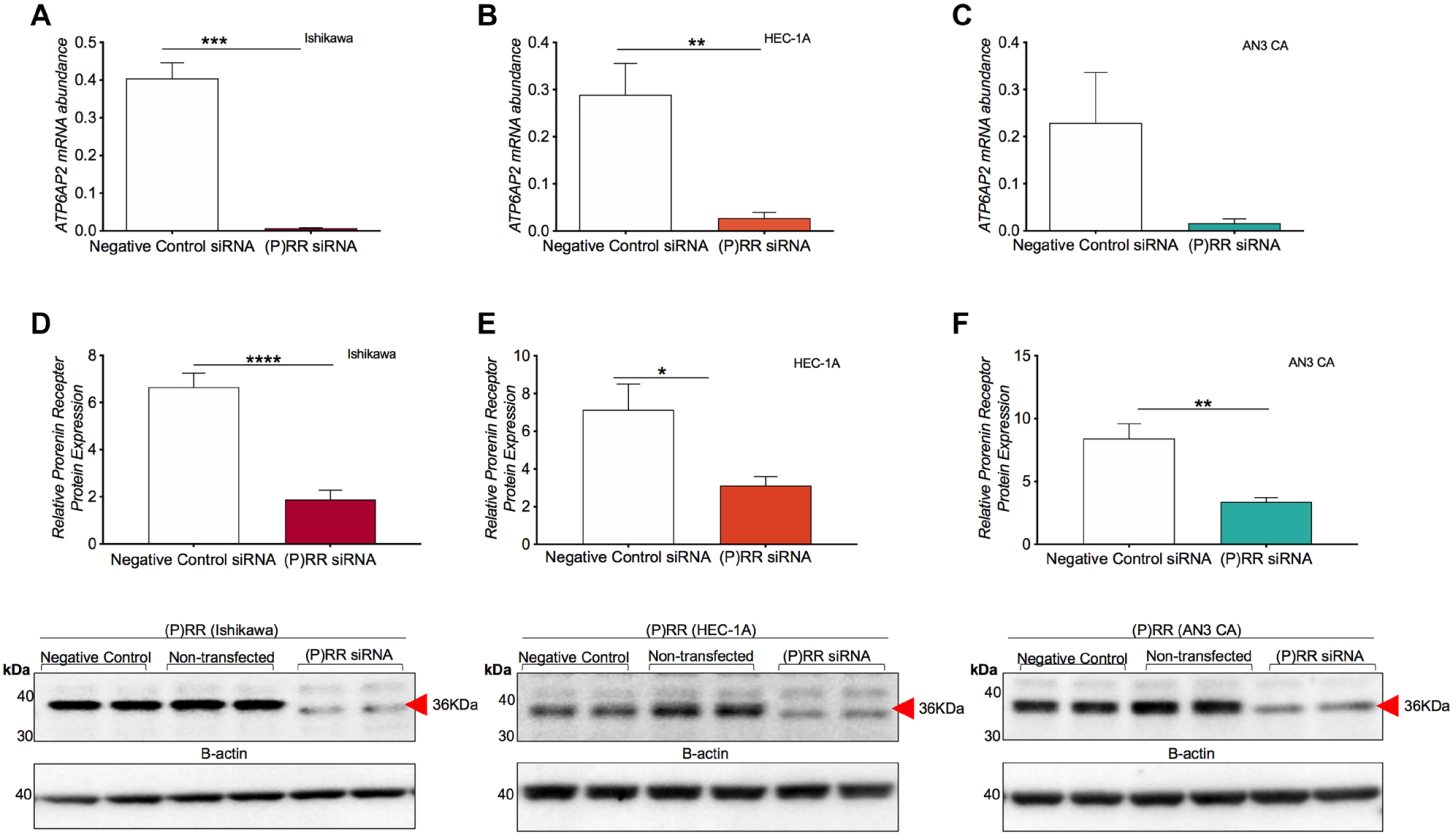
siRNA knockdown of ATP6AP2 gene and protein ((P)RR) expression in endometrial epithelial cancer cell lines. Messenger RNA expression of *ATP6AP2* following (P)RR siRNA transfection in Ishikawa (**A**), AN3CA (**B**) and HEC-1-A (**C**) cell lines. Complementary immunoblotting analysis of protein expression following transfection examining the Ishikawa (**D**), HEC-1-A (**E**) and AN3CA (**F**) cell lines. Mean ± SEM densitometric values are plotted in histograms as determined by pixel intensity analysis. ^*^ *P* < 0.05, ^**^ *P* < 0.01 and ^****^ *P* < 0.0001.

Transfection of Ishikawa and HEC-1-A cells with the (P)RR siRNA resulted in a greater than 89% reduction in *ATP6AP2* mRNA compared with the negative control siRNA (Ishikawa: *P* = 0.0003, HEC-1-A: *P* = 0.002; Figure 2A and 2B). The apparent decrease in *ATP6AP2* mRNA levels caused by the (P)RR siRNA in the AN3CA cell line was not statistically significant (AN3CA: *P* = 0.298; Figure 2C).

Transfection with the (P)RR siRNA also resulted in a significant knockdown of (P)RR protein (Figure 2D–2F). The Ishikawa cells saw a 72% decrease in (P)RR protein levels (*P* < 0.0001) while expression in the HEC-1-A (*P* = 0.011) and AN3CA (*P* = 0.002) cell lines decreased by 56% and 59%, respectively.

### Effect of ATP6AP2 siRNA: cellular viability and proliferation

**Figure 3:**
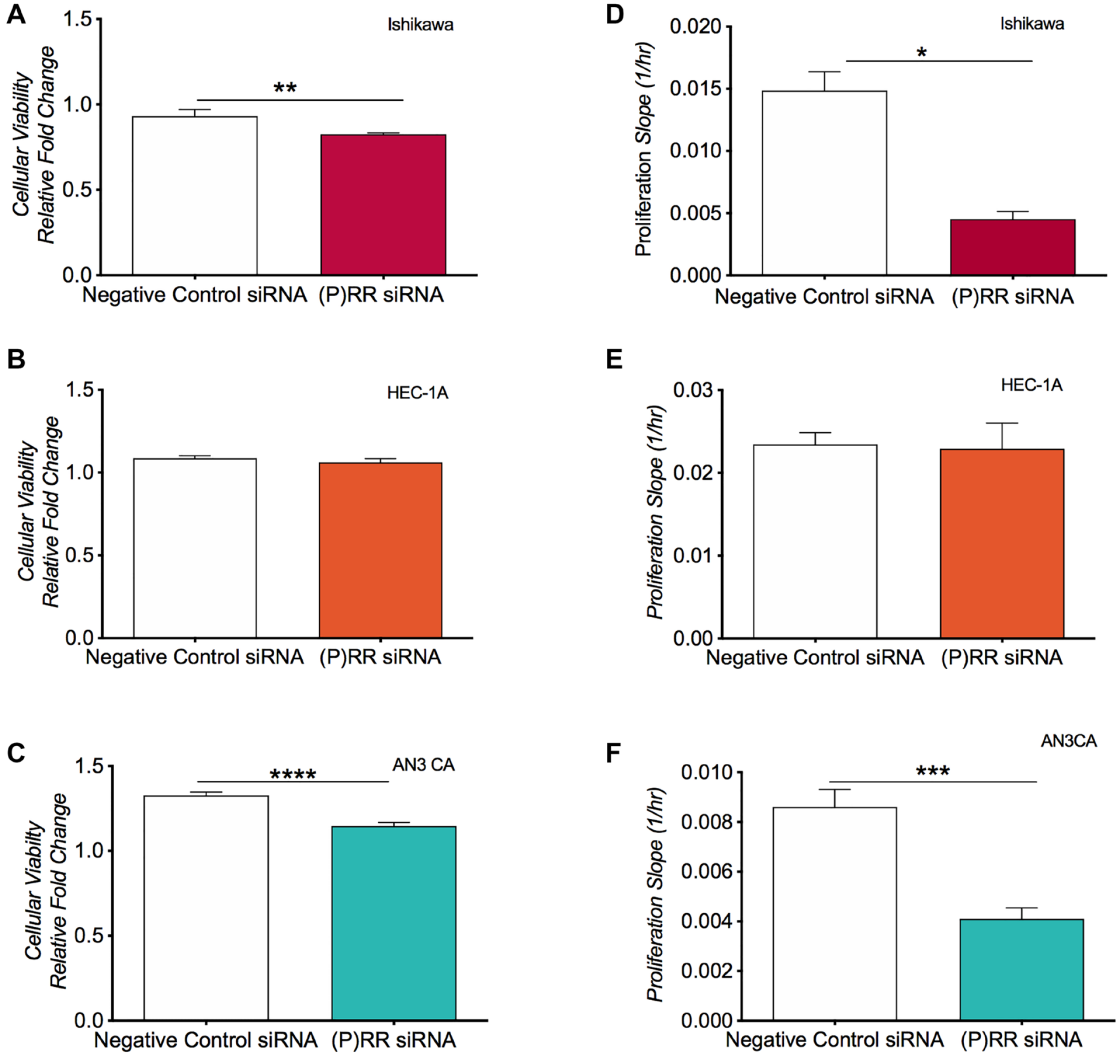
Analysis of cellular viability and cellular proliferation following siRNA knockdown of ATP6AP2 in the endometrial epithelial cancer cell lines. Cell viability was assessed using the fluorometric resazurin assay for the Ishikawa (**A**), HEC-1-A (**B**) and AN3CA (**C**) cell lines. Cell viability was determined as the mean ± SEM fluorescence intensity value measured as a relative conversion fold change of non-fluorescent blue to fluorescent pink dye (with excitation emission wavelengths of 530 nm and 590 nm). (**D**–**F**) Proliferative capacity was assessed using xCelligence technology whereby electrical impedance acted as a surrogate measure of a cell’s proliferative rate in the three cell lines. Mean ± SEM values are plotted in histograms. ^*^ *P* < 0.05, ^**^ *P* < 0.01, ^***^ *P* < 0.001 and ^****^ *P* < 0.0001.

Cell viability following (P)RR siRNA transfection was assessed via resazurin assay. In the Ishikawa and AN3CA cell lines (Figure 3A and 3C), (P)RR siRNA transfection resulted in a 11% and 33% decrease in cell viability by 48 h (*P* = 0.004 and *P* < 0.0001, respectively). (P)RR siRNA transfection had no effect on HEC-1-A cell viability (Figure 3B). Cell proliferation following (P)RR siRNA knockdown was assessed using the xCELLigence RTCA system. Similarly, siRNA knockdown of the (P)RR reduced the rate of in cell proliferation both in the Ishikawa (Figure 3D; *P* = 0.051) and AN3CA cell lines (Figure 3F; *P* = 0.0001) but had no effect in HEC-1-A cells (Figure 3E).

### siRNA knockdown of ATP6AP2: proteomics

TMT mass spectrometry-based proteomics was restricted to Ishikawa cells as they exhibited the highest expression of ATP6AP2 mRNA and achieved the greatest and most consistent knockdown following siRNA transfection.

**Figure 4:**
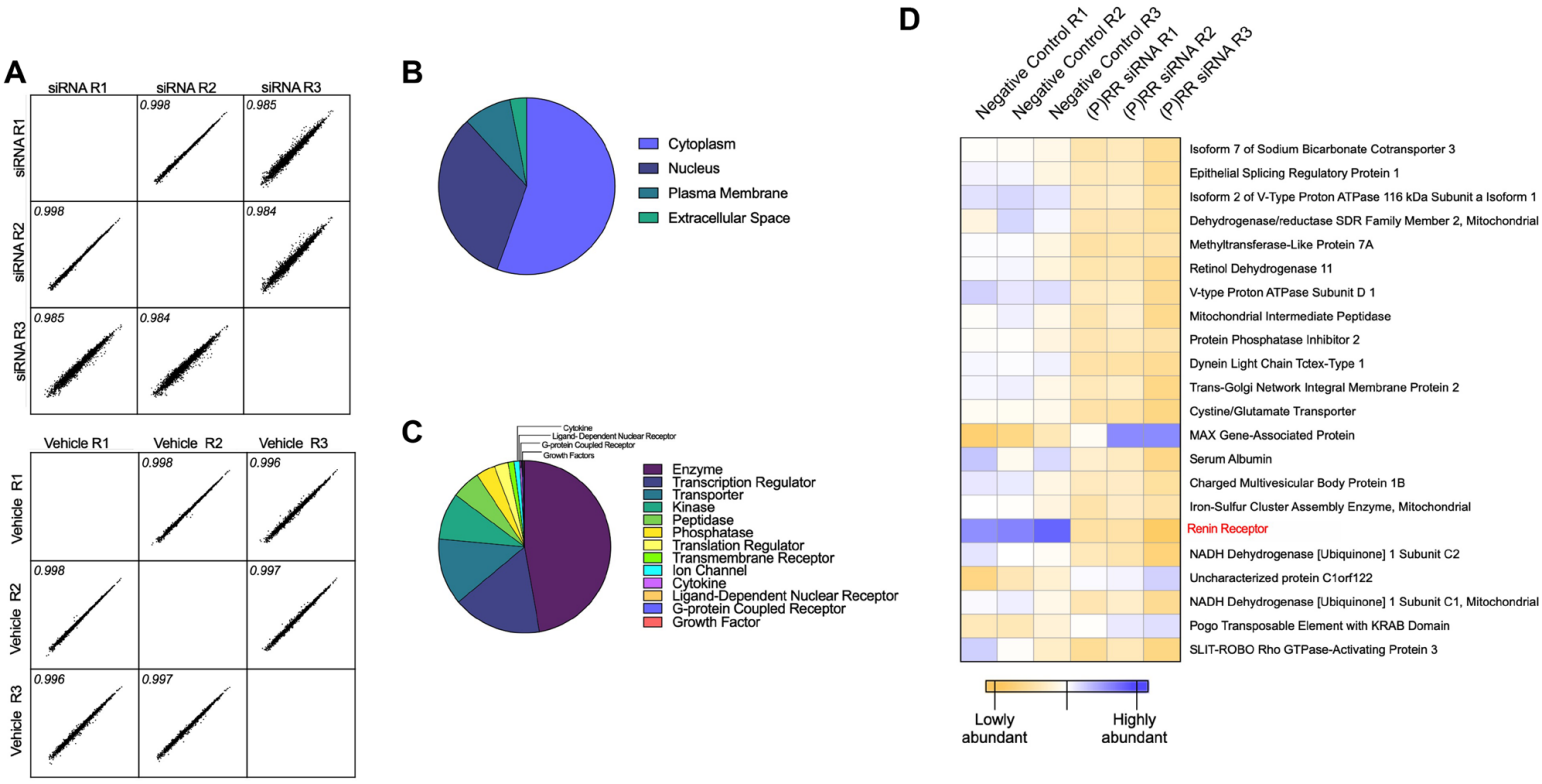
Proteomic assessment of the functional consequences of ATP6AP2 knockdown in the Ishikawa endometrial epithelial cancer cell line. Tandem Mass Tags (TMT) mass spectrometry-based proteomics was used to quantitatively assess differences in the protein composition of the Ishikawa cell line following siRNA knockdown of ATP6AP2. (**A**) Pearson multi-scatter plot displaying the correlation among the three biological replicates of the transfected (top) and control (bottom) samples. (**B**) Ingenuity Pathway Analysis (IPA) of the overall distribution and (**C**) protein class of each of the proteins identified. Scatter plots were constructed using Perseus (version 1.6.10.43) and the distribution and class assessment using licensed Ingenuity Pathway Analysis software (IPA^®^, Qiagen) and the Ingenuity Knowledge Base. (**D**) Quantitative expression profiles (heat map) indicated that all transfected samples cluster as do the vehicle control samples. In addition, the heat map displays the protein identifications with most significant abundance changes.

We identified a complex proteomic signature comprising a total of 5,241 proteins. An average of 10.9 peptide matches (encompassing 10 unique peptide matches) were generated per protein; representing an average peptide coverage of 29.1% per protein. The multi-scatter plot (Figure 4A) highlighted a strong correlation (Pearson ≥0.984) between each of the replicates. Preliminary interrogation of the global Ishikawa cell proteome by Ingenuity Pathway Analysis (IPA) indicated the overall distribution and protein class (Figure 4B and 4C) of the proteins identified. These proteins largely localized to the cytoplasm (~52%) and nucleus (~31%) and primarily belonged to the enzyme class of proteins (47%).

**Figure 5:**
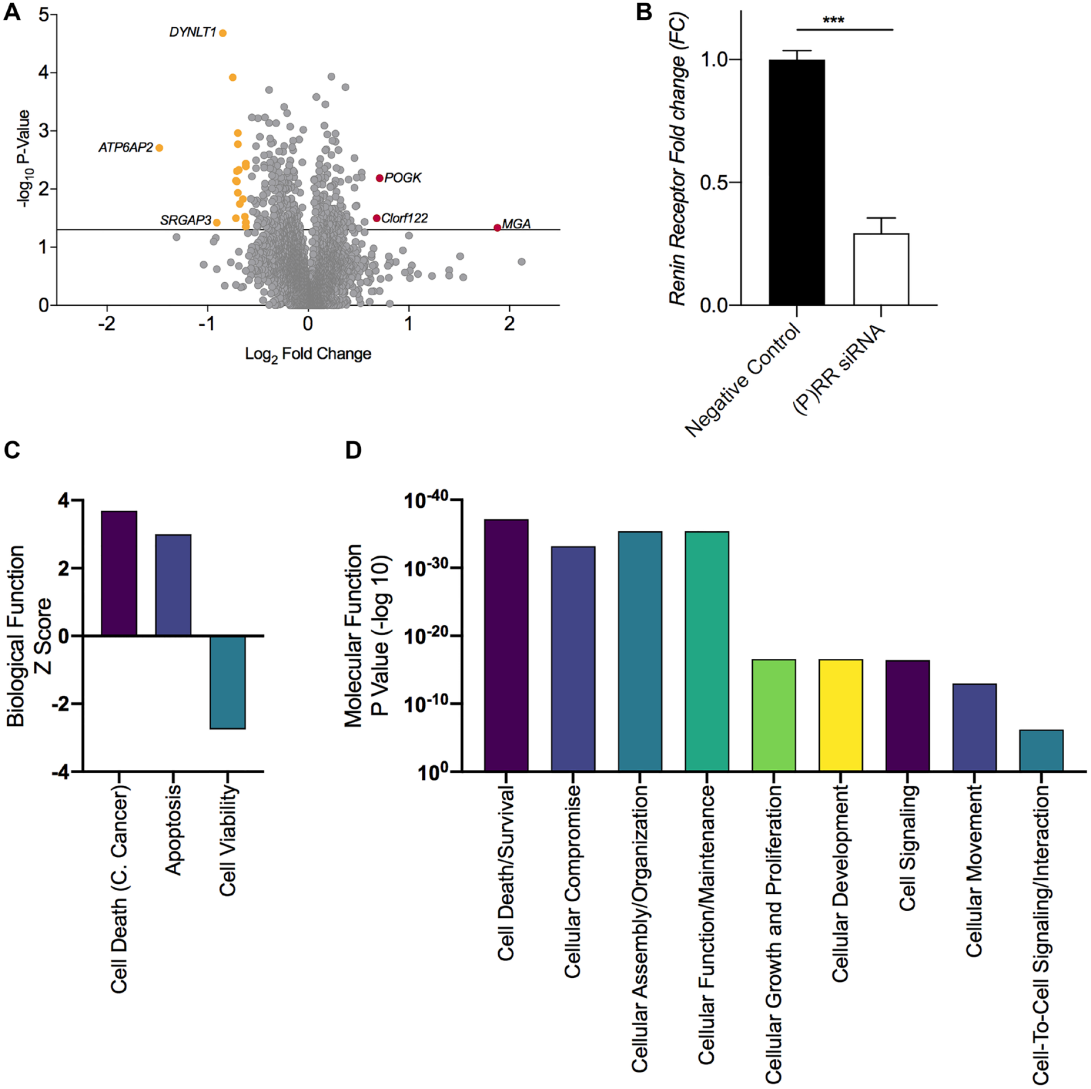
Proteomic assessment of the functional consequences of ATP6AP2 and Gene ontology based functional annotation. (**A**) Volcano plot displaying the total proteome and differentially expressed proteins following transfection; 3 proteins were upregulated (FC ≥ 1.5 and *P* ≤ 0.05) and 19 were down regulated (FC ≤ 0.667 and *P* ≤ 0.05). (**B**) Histogram confirming the loss of (P)RR expression following transfection. (**C**) Broad biological function and molecular and cellular function (**D**) as determined by licensed Ingenuity Pathway Analysis software (IPA^®^, Qiagen) utilizing the ‘canonical pathway, disease and function’ analyses, which returned *P*-values (an enrichment measurement based on the number of proteins that map to a particular pathway, function or regulator), and *Z*-score, which is a prediction scoring system that assesses activation or inhibition of a given pathway or function based upon statistically significant patterns in the dataset and prior biological knowledge previously manually curated in the Ingenuity Knowledge Base. C. Cancer = cervical cancer. Mean ± SEM values are plotted in the histogram. ^***^ *P* < 0.001.

Analysis of the proteomic profiles of each group ((P)RR siRNA vs. negative control siRNA) returned three proteins that were significantly upregulated (Fold Change (FC) ≥ 1.5 and *P* ≤ 0.05) and 19 that were significantly downregulated (FC ≤ 0.667 and *P* ≤ 0.667) (Figure 4D and Supplementary Table 1). The (P)RR was the most significantly downregulated protein in the (P)RR siRNA (*P* = 0.0006) transfected cell sample (Figure 4D and Figure 5A, 5B). The complete returned proteome is provided in Supplementary Table 2.

Additional IPA assessment on the basis of Gene Ontology implicated a predictive increase in the broader biological function categories (Figure 5C) of ‘Cell Death of Cervical Cancer Cell Lines’ (*P* = 5.72E-37, *z*-score 3.688), ‘Apoptosis’ (*P* = 1.6E-24, *z*-score 2.999) and a corresponding decrease in ‘Cell Viability’ (*P* = 2.2E-26, z-score −2.747) and ‘Cell Proliferation’ (*P* = 2.68E-17, *z*-score −3.895). Notable enrichment was also identified in the general molecular and cellular function categories (Figure 5D) of ‘Cell death and Survival’ (*P* = 6.97e-38) and ‘Cellular Growth and Proliferation’ (*P* = 2.68e-17).

### s(P)RR expression in endometrial epithelial cancer cell lines, patient plasma and uterine fluid samples

**Figure 6:**
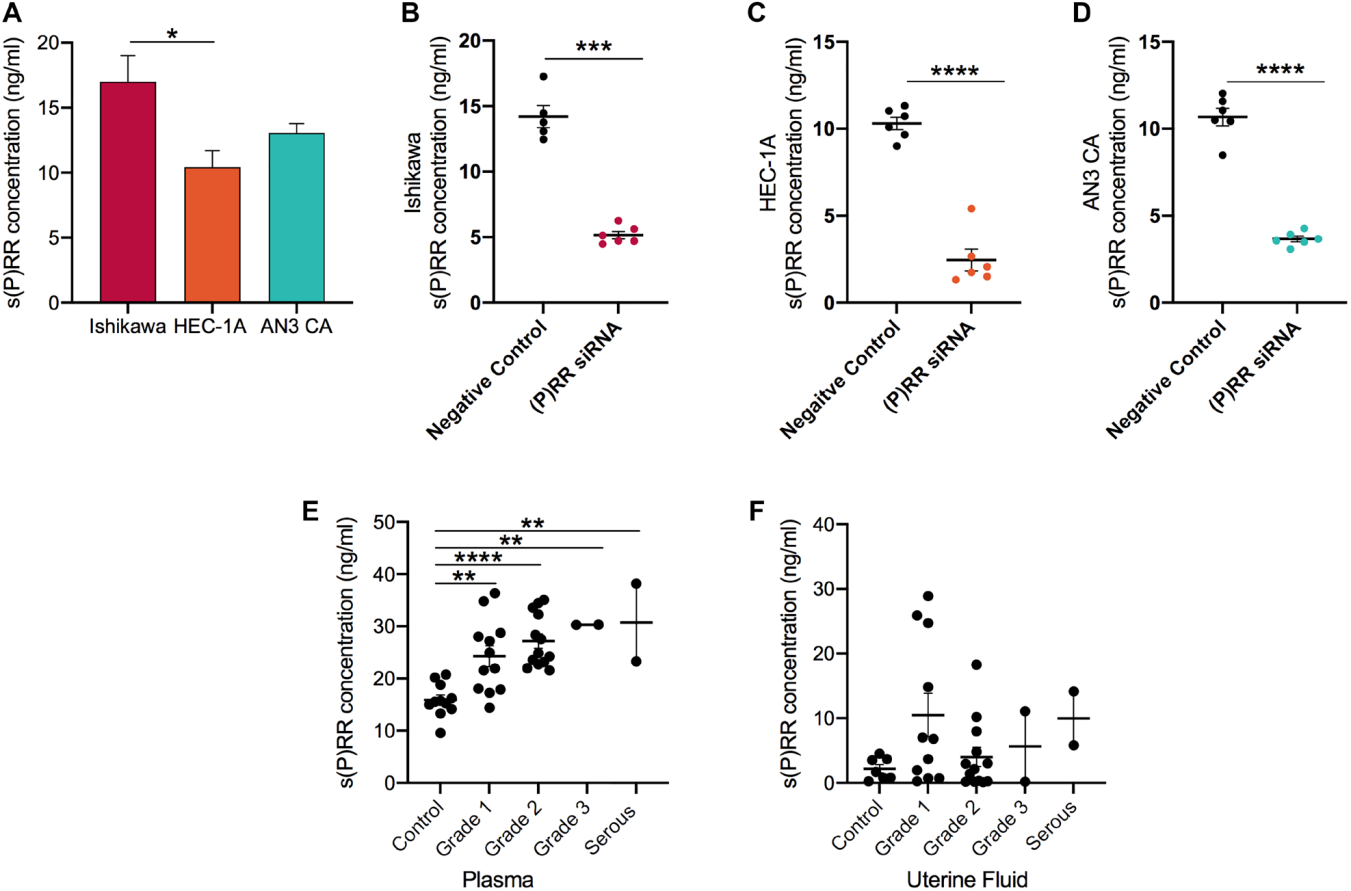
Soluble prorenin receptor (s(P)RR) expression in endometrial epithelial cancer cell culture supernatants and in endometrial cancer patient plasma and uterine fluid samples. Soluble (P)RR expression was measured by a commercially available human ELISA in three endometrial cancer cell lines prior to (**A**) and after transfection (**B**–**D**) with (P)RR siRNA. In addition, s(P)RR was measured in human plasma (**E**) and uterine fluid/lavage samples (**F**) donated by endometrial cell cancer patients. Mean ± SEM values are plotted in scatterplots. ^*^ *P* < 0.05, ^**^ *P* < 0.01 and ^***^ *P* < 0.0010.

Levels of s(P)RR in Ishikawa cell supernatants (16.99 ng/ml) were significantly higher than in supernatants from HEC-1-A cells (10.43 ng/ml; *P* = 0.012) and were equivalent to AN3CA cell supernatants (13.06 ng/ml; Figure 6A). Following siRNA transfection, a dramatic reduction (64–76%) in the secretion of s(P)RR by Ishikawa (*P* = 0.0003), HEC-1-A and AN3CA cells (both *P* < 0.0001; Figure 6B–6D) was evident when compared to the negative control siRNA.

Logically, levels of secreted s(P)RR were positively correlated with corresponding levels of membrane bound (P)RR within each cell line (Ishikawa: r = 0.7, *P* = 0.02; HEC-1-A: r = 0.7, *P* = 0.02; AN3CA: r = 0.8, *P* = 0.001; data not shown).

To extend this analysis, s(P)RR was measured in the blood and uterine fluid (Figure 6E and 6F) of patients diagnosed with endometrial cancer. These data indicate that s(P)RR levels were significantly higher (15.88 vs. ~28.12 ng/ml) in the plasma of patients with endometrial cancer (Grade 1: *P* = 0.0032, Grade 2: *P* < 0.0001, Grade 3: *P* = 0.0062, serous: *P* = 0.0047) than in age-matched controls. Furthermore, levels increased with increasing cancer grade (Grade 1 = 24.27 ng/ml, Grade 2 = 27.18 ng/ml, Grade 3 = 30.30 ng/ml, serous = 30.73 ng/ml). s(P)RR levels were uninformative when analysed via uterine fluid.

## DISCUSSION

Our data confirms that the (P)RR is important for endometrial cancer development, contributing to both its viability and proliferative capacity. Moreover, our quantitative proteomics approach uncovered several putative protein interactions and pathways that rely on (P)RR for disease progression and may represent novel therapeutic targets in the treatment of endometrial cancer. Finally, we contend that circulating s(P)RR levels may have substantial potential as a novel biomarker for cancer diagnosis and prognosis.

This study is the first to report the mRNA and protein expression of (P)RR in three endometrial epithelial cancer cell lines; Ishikawa, AN3CA and HEC-1-A. Notably, (P)RR mRNA and protein levels were independent of tumour grade, with the highest expression detected in Ishikawa cells (grade 1), followed by AN3CA cells (grade 3) and finally HEC-1-A cells (grade 2). These data confirm our previous observations that (P)RR mRNA and protein levels were uncoupled from tumour grade in primary endometrial tumour samples [5].

Our efforts to knockdown (P)RR expression were successful, with a >89% decrease in *ATP6AP2* mRNA and a 56–72% decrease in (P)RR protein levels relative to cells treated with the negative control siRNA. Our data agree with that of Shibayama et al. who demonstrated a decrease in pancreatic ductal adenocarcinoma (PDAC) cell number following targeted reduction of the (P)RR [4] and is also in accordance with that of Ohba et al. who found that the (P)RR was essential for the proliferation of human breast cancer cells *in vitro* [3]. In PDAC cells, a reduction in (P)RR levels resulted in the formation of DNA lesions that trigged apoptosis and a consequential reduction in total cell number [4]. While we do not yet know if the reduction in (P)RR in our study also stimulated apoptosis, our data indicate that the loss of the (P)RR led to a predictive increase in the broad gene ontology biological function categories related to cell death. Together, these data suggest an implicit role for the (P)RR in modulating endometrial epithelial cellular viability and proliferation.

In exploring the proteomic profiles of siRNA treated Ishikawa cells, our analysis indicated three proteins whose abundance was significantly upregulated and 19 which were significantly downregulated. Of the 22 dysregulated proteins, three (‘Pogo transposable element with KRAB domain’, ‘Uncharacterized protein C1orf122’ and ‘Serum Albumin’) could not be ascribed to a known/relevant function using already published literature and, as such, these were not explored any further.

The most upregulated protein identified was ‘MAX gene-associated protein’ (MGA), a dual-specificity transcription factor that regulates the expression of the MAX-network and T-box family target genes [15]. MAX (myc-associated factor X) interacting proteins (including MGA) function as transcription activators/repressors that regulate genes controlling cell proliferation [15]. In non-tumourigenic cells, a delicate balance exists between MAX and its binding partners (MGA and MYC) that dictates transcription of target genes through heterodimerization. Specifically, heterodimers of MAX and MGA antagonize MYC-dependent cell transformation and vice versa [16]. Therefore overexpression of MGA, caused by (P)RR knockdown, could disturb this balance, limiting the supply of MAX for MYC heterodimerization and antagonizing MYC-dependent cell processes; re-routing the tumour cell from a proliferative to a non-proliferative state [17].

The most down regulated protein identified was the ‘Renin receptor’ (ATP6AP2), thus confirming the efficacy and specificity of the siRNA knockdown. To determine how the remaining proteins could mediate the effect of (P)RR on cell proliferation or viability we manually curated our identifications into several functional clusters including those with roles in tumour cell deacidification and redox regulation.

Extracellular tumour acidity is correlated with cancer aggressiveness. In tumours, changes in the expression and/or activity of plasma membrane transporters that facilitate H^+^ efflux maintain a higher intracellular pH and lower extracellular pH [18]. In our study, four proteins implicated in deacidification processes were downregulated including ‘Isoform 7 of Sodium Bicarbonate Cotransporter 3′ (SLC4A7), two subunits required for the assembly of the ‘V-Type Proton ATPase’ (ATP6V0A1 and ATP6V0D1) and the (P)RR, which is also a component of a V-ATPase. SLC4A7 is implicated in the pathophysiology of breast cancer [19] and increased expression of V-ATPase correlates with cancer grade in human pancreatic intra-epithelial neoplasms and PDAC [20]. It is therefore conceivable that the reduction in SLC4A7, (P)RR, ATP6V0A1 and ATP6V0D1 is contributing to the decrease in proliferation that we observed.

Another consequence of the oncogene driven reprogramming of metabolic pathways is the increased production of reactive oxygen species (ROS), which must be countered by endogenous antioxidants to avoid cell cycle arrest and cellular senescence [21]. In this study, siRNA knockdown of (P)RR appeared to promote an environment that was not conducive to buffering the effects of ROS. Seven proteins with known roles in redox homeostasis were downregulated including ‘Cystine/Glutamate Transporter’ (SLC7A11), ‘Dehydrogenase/reductase SDR Family Member 2, Mitochondrial’ (DHRS2), ‘NADH Dehydrogenase 1 Subunit C1 (NDUFC1) and C2’ (NDUFC2), ‘Mitochondrial Intermediate Peptidase’ (MIPEP), ‘Epithelial splicing regulatory protein 1’ (ESRP1) and ‘Iron-Sulfur Cluster Assembly Enzyme’ (ISCU).

SLC7A11, is a sodium-independent pump and a key component of the system XC^−^ transporter (xCT), which mediates the exchange of intracellular glutamate for extracellular cystine, an essential precursor for glutathione (GSH) synthesis [22]. xCT is upregulated in many cancers promoting tumourigenesis through its antioxidant function [22]. As such, tumour cells are uniquely dependent on xCT [23]. Loss of xCT, as seen in our study, renders tumours hypersensitive to ROS and oxidative DNA damage so much so that they might succumb to a lethal accumulation of free radicals triggering an oxidative stress-induced cell death [24]. As an adjunct to this, down regulation of ESRP1 (enhances xCT function [25]), DHRS2 (protects against ROS-induced apoptosis [26]), NDUFC1/NDUFC2 (required for mitochondrial respiration and ATP generation [27]) and MIPEP (required for maturation of oxidative phosphorylation related proteins [28]), as seen in our study, may further exacerbate this situation.

How each of these proteins specifically relate to and/or interact with the (P)RR remains to be fully clarified. However, a preliminary analysis of the 22 dysregulated proteins returned two putative protein-protein interaction clusters. The largest cluster demonstrated a proven interaction between *ATP6AP2* and the ATP6V0D1 (STRING combined score 0.979), which itself, through several intermediates, interacted with NDUFC1, NDUFC2, ISCU and MIPEP, thereby bridging the deacidification and redox regulator capacities outlined above. Further work is required to fully elucidate these interactions and to better understand the consequences of (P)RR loss/reduction.

Collectively, our data indicate that targeting the (P)RR by an siRNA approach (such as in this study) or with an alternative anti-(P)RR monoclonal antibody approach currently being explored by Wang et al. [29] may be a viable therapeutic strategy against endometrial cancer. Indeed, our data suggest that its loss may significantly mitigate disease risk by reducing the expression of several proteins (ESRP1 and DHRS2) with known deleterious effects in endometrial cancer (Supplementary Table 3) in addition to modulating cell viability and proliferative capacity. This idea is further supported by a host of literature whereby the (P)RR has been shown to promote several types of cancers [3, 4, 29–32].

In addition, it has now been suggested that (P)RR expression or s(P)RR levels may serve as an adjuvant marker, in combination with current cancer-related protein markers, to aid diagnosing as well as predicting the severity and prognosis of various cancers. Shibayama et al. for example, indicated that s(P)RR is significantly elevated in the plasma of patients with PDAC when compared to healthy individuals [4]. This study demonstrates that s(P)RR levels in plasma are significantly higher in patients with endometrial cancer than in age-matched controls. Importantly, levels increased with increasing cancer grade. Thus s(P)RR in the blood could hold considerable value as a predictive or diagnostic test for patients with endometrial cancer.

In conclusion, our data indicate that the (P)RR is intimately involved in endometrial cancer growth and viability, whereby a reduction in (P)RR levels was associated with a significant reduction in cellular proliferation and viability. This is putatively mediated by the sequential reduction of proteins such as MGA, SLC4A7, SLC7A11 or DHRS2. We therefore contend that knocking down or inhibiting the (P)RR could be a novel therapeutic strategy for the treatment of endometrial cancer. Moreover, s(P)RR levels in the blood, may have substantial potential as a novel biomarker for cancer diagnosis and prognosis prediction going forward.

## MATERIALS AND METHODS

### Materials

All chemicals were purchased from Sigma-Aldrich (St Louis, MO, USA) unless otherwise stated.

### Patient samples

Uterine fluid (collected as previously described [33]) and plasma were collected from patients (37–50 years of age) with or without endometrial cancer. The use of these samples was approved by the Hudson Research Institute, Melbourne Human Research Ethics Committee (HREC #02031B and HREC#06032C). A total of 36 samples of uterine fluid and plasma were collected (7 control, 11 Grade 1, 13 Grade 2, 3 Grade 3 and 2 serous cancer samples) meeting the International Federation of Gynecology and Obstetrics developed grading system.

### Cell culture

Ishikawa (histological grade 1), AN3CA (histological grade 3) and HEC-1-A (histological grade 2) cell lines, were acquired from the American Type Culture Collection (ATCC; Manassas, VA, USA) and approved by the University of Newcastle Human Research Ethics Committee (H-2020-0398). Ishikawa and AN3CA cells were cultured in minimum essential media (MEM) supplemented with 5% heat inactivated fetal bovine serum (HI-FBS, Bovogen Biologicals, Victoria, Australia), and 1% antibiotic-antimycotic (Gibco, Waltham, MA, USA). HEC-1-A cells were maintained in McCoy’s 5A media supplemented with 10% HI-FBS, 1% antibiotic-antimycotic, and 2 mM L-glutamine (Gibco). All cells were incubated at 37°C with 5% CO_2_. Once confluent, cells were plated at 1.5 × 10^4^ cells/well in 6 well plates with 2 ml of growth medium without antibiotics for 24 h to form a monolayer.

### siRNA transfection

An *ATP6AP2* siRNA (Life Technologies, Carlsbad, CA, USA) was introduced to the cells using Lipofectamine^®^ 2000 Transfection Reagent (Invitrogen) and Opti-MEM (Thermo Fisher Scientific, Waltham, MA USA) according to the manufacturer’s recommendations. Briefly, cells were seeded as described above and incubated overnight to allow adherence. Cells were then transfected with 125 nM ATP6AP2 Stealth RNAi™ siRNA ((P)RR siRNA; identification number: HSS115475; catalog number: 1299001), 10 nM of Stealth RNAi™ siRNA Negative Control (catalogue number: 12935300) or vehicle (transfection reagents *sans* siRNA). After 24 h the medium was replaced and incubated for a further 24h after which, the supernatant and cell pellet were collected, snap frozen and stored at −80°C until required.

### Real-time reverse transcriptase polymerase chain reaction (qRT-PCR)

Total RNA was extracted using a RNeasy mini kit (Qiagen, Hilden, Germany) and immediately reverse transcribed using a SuperScript™ III Reverse Transcriptase kit with random hexamers (Invitrogen). RNA concentration and purity were determined using a Nanodrop ND-1000 spectrophotometer and RNA integrity was analyzed by gel electrophoresis.

qPCR was performed using an Applied BioSystems 7500 Real Time PCR system (Applied BioSystems, CA, USA). Sample were run in duplicate with the appropriate controls (-RT and dH_2_O) and contained 5 μl of SYBR Green PCR master mix, primers (Supplementary Table 4) and 10 ng of cDNA (10 μl total volume). Messenger RNA abundance (2^−ΔΔCT^) was calculated relative to the geometric mean of the housekeeping genes, β-actin (ACTB), 18S ribosomal 1 (RNA18S1), and Tyrosine 3-Monooxygenase/Tryptophan 5-Monooxygenase Activation Protein Zeta (YWHAZ) and compared with an internal control sample (term human placenta), which was incorporated into each run.

### NuPAGE and immunoblotting

Total protein was extracted using radio-immunoprecipitation assay (RIPA) lysis buffer containing 1% phenylmethylsulfonyl fluoride (PMSF). 10 μg of protein lysate was prepared in lithium dodecyl sulfate (LDS) sample buffer and sample reducing agent (NuPAGE; Thermo Fisher Scientific) before being resolved on pre-cast 4–12% NuPAGE BIS-Tris gels (Thermo Fisher Scientific). Proteins were then transferred to a polyvinylidene difluoride (PVDF) membrane. The membrane was blocked in 5% skim milk/5% bovine serum albumin (BSA) in Tris-buffered saline containing 0.1% Tween (TBST) at 4°C overnight. Membranes were incubated with a (P)RR primary antibody (ab40790; Abcam (Cambridge, England, UK); 1:1000, 2 h), washed thrice in TBST and incubated in anti-rabbit secondary antibody (Abcam; 1:5000, 1 h). Membranes were subsequently stripped (with 0.2M NaOH) and re-probed with anti-β-actin (1:5000, 1 h). Membranes were developed using the enhanced chemiluminescence detection kit (GE Healthcare, Chicago IL, USA) and imaged using an Amersham 600 imager (GE Healthcare). Densitometric analysis was achieved using Image J (National Institute of Health, Bethesda, MD, USA). The density of each band was corrected for β-actin as the loading control and further normalised to an internal control sample (pooled term amnion collected at caesarean section) on each membrane. Samples were run in duplicate and averaged for the final analysis.

### Cell viability and cellular proliferation

For the analysis of cell viability and proliferation, each cell line was seeded at a density determined by their growth rate patterns (Ishikawa: 1.5 × 10^3^; HEC-1-A: 5 × 10^3^; AN3CA: 2.5 × 10^2^). These seeding densities ensured 70–80% confluence of cells during the cell viability and proliferation assays.

Cell viability was determined using a resazurin assay. Five hours prior to the end of the transfection incubation, 20 μl of resazurin (Biovision, Milpitas, CA, USA) was added to each well and at 48 h the fluorescence intensity was measured using a Fluostar Optima (BMG Labtech, Ortenberg, Germany: excitation 540 nm and emission: 590 nm).

Cell proliferation was evaluated using an xCELLigence RTCA DP instrument (ACEA Biosciences, San Diego, CA, USA) [34]. The E-plate was first equilibrated with 100 μl of media and a background impedance score recorded over a 30 min period. Cells were then seeded in an additional 100 μl of media and incubated overnight. The medium was then replaced with experimental medium (200 μl) containing either the (P)RR siRNA, negative control siRNA or vehicle and incubated for 48 h with cell index measurements taken at 15 min intervals. The rate of proliferation was determined by calculating the slope of the cell index over 48 h.

### Enzyme linked immunosorbent assay (ELISA)

An s(P)RR ELISA (IBL, Switzerland; Catalogue #27782) was used to measure s(P)RR levels in patient plasma and uterine fluid samples (diluted 1:10 and 1:2, respectively) and pre-collected cell culture supernatant (1:5) as per the manufacturer’s instructions. The average of the duplicates (normalized for blank) and the standard curve was used to calculate the final sample concentration of s(P)RR (pg/mL).

### Proteomics: sample preparation and nLC-MS/MS analysis

Tandem mass tags (TMT; Thermo Fisher Scientific) were utilized to facilitate comparative and quantitative analyses (TMT 10 plex labels; negative control reps - 127N, 127C, 128N; (P)RR siRNA reps - 128C, 129N, 129C) [35, 36]. Digestion, TMT labeling efficiency and mixed 1:1 ratios were determined by LC-MS/MS, after a modified multi-dimensional strategy was used to enrich for the proteome [37]. The enriched proteome was subjected to offline hydrophilic interaction liquid chromatography (HILIC), and then analyzed using high resolution nano liquid chromatography tandem MS (nLC-MS/MS). Reverse phase nLC-MS/MS was performed on 12 HILIC enriched fractions using a Q-Exactive Plus hybrid quadrupole-Orbitrap MS coupled to a Dionex Ultimate 3000RSLC nanoflow high-performance liquid chromatography system (Thermo Fisher Scientific). Samples were loaded onto an Acclaim PepMap100 C18 75 μm × 20 mm trap column (Thermo Fisher Scientific) for pre-concentration and online de-salting. Separation was achieved using an EASY-Spray PepMap C18 75 μm × 500 mm column (Thermo Fisher Scientific), employing a linear gradient of acetonitrile (5–40%, 300 nl/min, 115 min). A Q-Exactive Plus MS System was operated in full MS/data dependent acquisition MS/MS mode (data-dependent acquisition). The Orbitrap mass analyzer was used at a resolution of 70,000, to acquire full MS with an m/z range of 380–2000, incorporating a target automatic gain control value of 1 × 10^6^ and maximum fill times of 50 ms. The 20 most intense multiply charged precursors were selected for higher-energy collision dissociation fragmentation with a normalized collisional energy of 32. MS/MS fragments were measured at an Orbitrap resolution of 35,000 using an automatic gain control target of 5 × 10^5^ and maximum fill times of 120 ms.

### Data processing and analysis

Database searching was performed using Proteome Discoverer 2.4 (Thermo Fisher Scientific). SEQUEST HT was used to search against the UniProt Human database (42,307 sequences, downloaded 12th November 2019). Database searching parameters included up to two missed cleavages, precursor mass tolerance of 10ppm and fragment mass tolerance of 0.02Da. trypsin was designated as the digestion enzyme. Cysteine carbamidomethylation (C) was set as a fixed modification; dynamic modifications included acetylation (N-Terminus), oxidation (M), phospho (S/T and Y) and TMT6plex (K, N-Terminus). Interrogation of the corresponding reversed database was performed to evaluate the false discovery rate of peptide identification using Percolator on the basis of q-values. A fixed false discovery rate of 1% was set at the peptide level. The list of proteins was refined to include only those with a quantitative value in all three replicates, and a minimum of two unique peptides. Perseus version 1.6.10.43 was used to generate the scatter plots and heatmap. Ingenuity Pathway Analysis software (IPA^®^, Qiagen) was used to analyze the refined proteomic list as previously described [35]. Canonical pathway, disease and function analyses were assessed using the returned *P*-values and *Z*-scores [38]. A STRING (Search Tool for the Retrieval of Interacting Genes/Proteins) analysis (https://string-db.org/; accessed 13th July 2020) was conducted to examine putative protein-protein interactions (Supplementary Figure 1).

### Statistical analysis

Statistical significance was determined using the GraphPad Prism statistical software (version 8.2.1, San Diego, USA) using one-way and two-way ANOVAs, multiple comparisons by Tukey’s multiple comparison test and an unpaired *t*-test. Differences with a value of *P* ≤ 0.05 were considered statistically significant. Each cell experiment was conducted using a minimum of three biological and three technical replicates. All data are expressed as means ± S.E.M.

## SUPPLEMENTARY MATERIALS







## Abbreviations

(P)RR: prorenin receptor
RAS: renin angiotensin system
FIGO: International Federation of Gynecology and Obstetrics
ATCC: American Type Culture Collection
s(P)RR: soluble prorenin receptor
TMT: tandem mass tags
HILIC: hydrophilic interaction liquid chromatography
nLC-MS/MS: nano liquid chromatography tandem MS
IPA: Ingenuity Pathway Analysis software
STRING: Search Tool for the Retrieval of Interacting Genes/Proteins)
FC: Fold Change
GO: Gene Ontology
PDAC: pancreatic ductal adenocarcinoma
