# Supplementary material for: Role of the prorenin receptor in endometrial cancer cell growth
Source: Oncotarget. 2022 Apr 1;13:587–99. doi: 10.18632/oncotarget.28224 (PMC8986267; doi:10.18632/oncotarget.28224)
Supplement: Supplementary file 1 [file oncotarget-13-28224-s001.pdf]

Role of the prorenin receptor in endometrial cancer cell growth

SUPPLEMENTARY MATERIALS

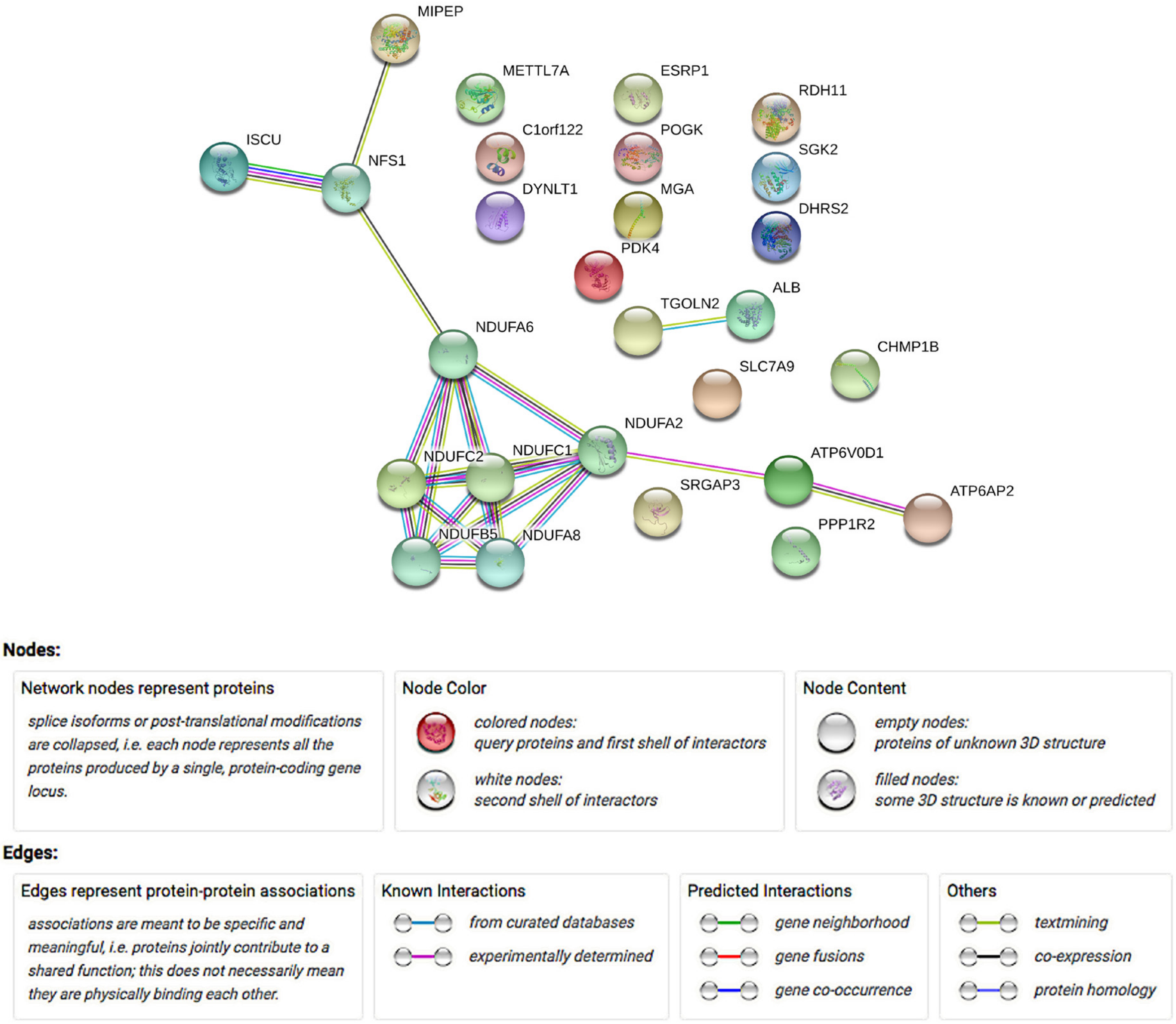

Supplementary Figure 1: STRING analysis of the proteomic dataset returned following Tandem Mass Tags (TMT) mass spectrometry-based proteomics. The 22 dysregulated proteins following TMT mass spectrometry following siRNA knockdown were interrogated for putative protein-protein interactions using the online STRING (Search Tool for the Retrieval of Interacting Genes/Proteins) platform (https://string-db.org/; accessed 13.07.20). This analysis returned two putative interaction clusters that arise from computational prediction, knowledge transfer between organisms, and/or from interactions aggregated from other primary databases (covering 24,584,628 proteins from 5090 organisms).

Supplementary Table 1: Total Tandem Mass Tags (TMT)-labelled endometrial cancer proteome, (P)RR siRNA and negative control siRNA treated Ishikawa cells

Accession	Description	*t* Test	Log2 Fold change
Q8IWI9	MAX gene-associated protein	0.046267327	1.880
Q9P215	Pogo transposable element with KRAB domain	0.006472898	0.710
Q6ZSJ8	Uncharacterized protein C1orf122	0.031790132	0.680
Q9Y6M7-7	Isoform 7 of Sodium bicarbonate cotransporter 3	0.003628848	−0.620
Q6NXG1	Epithelial splicing regulatory protein 1	0.044429079	−0.620
P41236	Protein phosphatase inhibitor 2	0.004073581	−0.620
O43493	Trans-Golgi network integral membrane protein 2	0.037525067	−0.620
Q99797	Mitochondrial intermediate peptidase	0.029819245	−0.630
Q7LBR1	Charged multivesicular body protein 1b	0.014874532	−0.650
O95298	NADH dehydrogenase [ubiquinone] 1 subunit C2	0.018008751	−0.680
Q9H1K1	Iron-sulfur cluster assembly enzyme ISCU, mitochondrial	0.004685317	−0.690
Q93050-1	Isoform 2 of V-type proton ATPase 116 kDa subunit a isoform 1	0.001091984	−0.700
Q9UPY5	Cystine/glutamate transporter	0.001693745	−0.700
O43677	NADH dehydrogenase [ubiquinone] 1 subunit C1, mitochondrial	0.011606073	−0.700
Q8TC12	Retinol dehydrogenase 11	0.007382326	−0.710
P61421	V-type proton ATPase subunit d 1	0.004917348	−0.710
Q13268	Dehydrogenase/reductase SDR family member 2, mitochondrial	0.007202643	−0.720
P02768	Serum albumin	0.031589381	−0.720
Q9H8H3	Methyltransferase-like protein 7A	0.000120546	−0.750
P63172	Dynein light chain Tctex-type 1	2.07236E-05	−0.850
O43295	SLIT-ROBO Rho GTPase-activating protein 3	0.037883627	−0.910
O75787	Renin receptor	0.001951605	−1.480

This table contains the complete proteomic profile achieved using TMT-based mass spectrometry along with the appropriate protein FDR confidence value, unique UniProt Human accession number, number of unique peptides, raw fold change value and log2 transformed fold change value, and the scaled abundances score for each peptide. A legend of essential parameters is also included with the document.

Supplementary Table 2: The complete list of dysregulated proteins following siRNA knockdown and Tandem Mass Tags (TMT) mass spectrometry-based proteomics. See Supplemetnary Table 2

Supplementary Table 3: Protein Atlas Summary Table. See Supplementary Table 3

Supplementary Table 4: Quantitative real time PCR primers summary

Gene	GeneBank Accession No.	Primer Sequence (5′–3′)	Concentration	Annealing temperature
ATP6AP2	NM_005765	**F**: ACAATGAAGTTGACCTGCTCTTTCTTTCTG<br>**R**: CCTTGGCTAGATGCTTATGACGAGACA	100 nM	76°C
RNA18S1	M10098.1	**F**: GTAACCCGTTGAACCCCATT<br>**R**: CCATCCAATCGGTAGTAGCG	100 nM	81°C
ACTB	NM_001101	**F**: CGCGAGAAGATGACCCAGAT<br>**R**: GAGTCCATCACGATGCCAGT	100 nM	83°C
YWHAZ	NM_001135699	**F**: CCTGCATGAAGTCTGTAACTGAG<br>**R**: GACCTACGGGCTCCTACAACA	100 nM	76°C

This table contains the GeneBank accession number, primer sequence, concentration and annealing temperature for each primer set utilized in this study. Each sample was run in duplicate with the appropriate controls (-RT; dH2O). Messenger RNA abundance ($2^{-\Delta\Delta CT}$) was calculated relative to the genomic mean of the following housekeeping genes; β-actin (ACTB), RNA, 18S ribosomal 1 (RNA18S1) and Tyrosine 3-Monooxygenase/Tryptophan 5-Monooxygenase Activation Protein Zeta (YWHAZ), and compared with a calibrator sample (term human placenta) which was included into each run.
